# Sport Satisfaction and Psychological Well-Being in Collegiate Athletes: The Role of Upbringing, Athletic Status, and Adaptive Psychological Attributes

**DOI:** 10.3390/sports14060222

**Published:** 2026-05-28

**Authors:** Akorede A. Teriba, Radomir R. Mitic, Kathryn M. Ellingson, Amber M. Peterson, Aaron M. Cooper, Andrew C. Lenway, Cassidy M. Brown, Henry Rott, Jimmy J. Morin

**Affiliations:** 1 Department of Education, Health & Behavior Studies, University of North Dakota, 231 Centennial Drive, Grand Forks, ND 58202, USA; 2 School of Education, William & Mary, 301 Monticello Avenue, Williamsburg, VA 23185, USA; rrmitic@wm.edu

**Keywords:** grit, growth mindset, emotion regulation, self-compassion, sport satisfaction

## Abstract

Athletes face a variety of pressures related to their sport participation, and these demands can contribute to persistent mental health challenges. The aim of this study was to examine the role of grit, growth mindset, emotion regulation, self-compassion, and psychological well-being in collegiate athletes’ sport satisfaction. Participants (*N* = 263) were recruited through CloudResearch and outreach emails to athletic programs. The sample included individuals from 43 U.S. states and represented rural, suburban, and urban communities. Among the 30 sports represented, basketball, football, and soccer had the highest participation. Results indicated significant differences in sport satisfaction (*p* = 0.007, η^2^ = 0.04) and growth mindset (*p* = 0.017, η^2^ = 0.03) across communities of upbringing, as well as differences in sport satisfaction across years in college (*p* = 0.008, η^2^ = 0.06). Scholarship status was associated with significant differences in sport satisfaction (*p* < 0.001, *d* = 0.85) and expressive suppression (*p* = 0.019, *d* = 0.31). Cognitive reappraisal (*r* = 0.427) demonstrated the strongest association with psychological well-being, whereas growth mindset (*r* = 0.501) showed the strongest association with sport satisfaction. Additionally, a significant interaction effect emerged between growth mindset and psychological well-being (*p* = 0.033, Δ*R*^2^ = 0.01) in predicting sport satisfaction. These findings highlight the importance of supporting student-athletes in effectively regulating their emotions and maintaining a belief in their capacity for growth, as both factors appear critical for promoting psychological well-being and enhancing satisfaction with the athletic experience.

## 1. Introduction

Intercollegiate athletics in the United States represent a widely popular form of entertainment for the general public. However, for the more than 550,000 National Collegiate Athletics Association (NCAA) student-athletes [1], the college experience involves far more than performance highlights and competitive success. Research has raised concerns about troubling trends in student-athletes’ mental health, with estimates indicating that at least 33% of collegiate athletes experience symptoms of depression [2,3,4]. Athletes face a variety of pressures related to sport participation, and these demands can contribute to persistent mental health challenges [5].

Financial compensation, or lack thereof, has also been shown to relate to student-athletes’ stress and overall mental health [6]. The majority of athletic compensation has consisted of partial scholarships, with approximately 1% of collegiate athletes receiving full funding and fewer than 2% of high school student-athletes seeking college admission receiving athletic scholarships [7]. Subsequent to data collection for this study, updates to collegiate athlete compensation policies following the 2025 settlement of *House v. NCAA* replaced scholarship caps with roster limits, thereby increasing the number of scholarships available to collegiate athletes [8]. Those who receive financial compensation may also experience heightened pressure related to reputation, performance expectations, and fear of losing their support [9].

Collegiate student-athletes frequently report that their athletic experience can exacerbate existing mental health difficulties [10]. The combination of intense competition and limited autonomy over daily life has been linked to physical and emotional exhaustion, depersonalization, reduced social connections, and negative self-perception, factors that collectively result in burnout [11]. Additionally, anti-rest culture and heightened public expectations can lead to feelings of overwhelm, particularly when athletes are restricted from engaging in activities that provide social support [12].

Athletes’ experiences are also shaped by their broader social environments. Community of upbringing, defined as the type of locality in which an individual is raised (i.e., rural, suburban, urban), reflects the environmental contexts that influence access to resources, social networks, and opportunities [13,14]. Differences across these contexts have been associated with variability in developmental outcomes, including access to support systems and availability of community resources [15,16]. As a result, athletes from different communities may enter collegiate sport with distinct psychological frameworks and experiences that shape both their well-being and satisfaction.

Athletes who demonstrate resilience are better able to withstand the mental health challenges associated with athletic participation [17]. Stakeholders in athletes’ lives (e.g., coaches, trainers, and parents) often assume that personal growth naturally emerges from adversity and that greater adversity is inherently beneficial. However, these same stakeholders may underestimate the importance of the psychological attributes that enable athletes to grow from adversity [18]. In reality, an athlete’s capacity for growth depends on their ability to effectively manage pressure and cultivate an optimistic, growth-oriented mindset, both of which support psychological well-being [19].

## 2. Psychological Well-Being in Student-Athletes

The World Health Organization describes mental health as an individual’s ability to manage their daily stressors and work productively to contribute to their community [20]. Psychological well-being is a core component of overall health and exists along a continuum ranging from negative mental states (e.g., depression) to positive mental states (e.g., life satisfaction) [21]. Individuals with high psychological well-being tend to experience a sense of autonomy and purpose, emotional well-being, and feelings of social acceptance [22].

However, various life demands and challenges can hinder an individual’s ability to achieve and maintain psychological well-being. This study examines psychological attributes that may support individuals in managing these challenges while maintaining well-being. Specifically, we focus on grit, growth mindset, emotion regulation (i.e., cognitive reappraisal, expressive suppression), and self-compassion as key mechanisms underlying persistence and adaptation in the face of difficulty [23].

## 3. Grit and Growth Mindset in Sport

Grit, defined as perseverance and passion for long-term goals [24], has been shown to help individuals cope with negative affect following challenging stressors [25]. Individuals with higher levels of grit tend to engage more consistently and effortfully in sport-related activities, including training, competition, and decision-making [26]. Among student-athletes, grit has been positively associated with self-efficacy, life satisfaction, academic performance, and reduced anxiety [27]. Additionally, environments that foster perseverance may contribute to the development of grit among athletes [28].

Implicit theory explains how individuals perceive the malleability of their abilities, distinguishing between fixed (entity) and growth (incremental) mindsets. A growth mindset reflects the belief that abilities can be developed through effort [29] and has been linked to positive outcomes such as academic success, healthy behavioral patterns, and life satisfaction across socioeconomic contexts [30,31]. In contrast, individuals with a fixed mindset may become discouraged when faced with challenges, as they perceive their abilities as static.

Within sport, a growth mindset enables athletes to adapt to challenges and persist through adversity. Athletes who adopt this perspective are more likely to find meaning in their athletic pursuits and maintain motivation through setbacks [32,33]. They also tend to be future-oriented, emphasizing continuous improvement [34], which can enhance both individual resilience and team dynamics. Conversely, athletes with a fixed mindset may struggle during demanding training and competition, limiting both performance and satisfaction [35].

## 4. Emotion Regulation and Self-Compassion

Emotion regulation is increasingly recognized as a critical factor in athletes’ performance and psychological functioning [36]. Research suggests that emotion regulation strategies provide benefits beyond cognitive techniques alone, as approaches that neglect emotional processes may inadvertently increase focus on unwanted thoughts [37]. Deficits in emotion regulation have been linked to impaired functioning and increased risk of psychological disorders [38]. Accordingly, improving emotion regulation may play an important role in enhancing athletes’ well-being.

Athletes are encouraged to use a range of strategies to manage their emotions before, during, and after competition [39]. These strategies may include both simple techniques (e.g., distraction) and more complex approaches (e.g., meaning making) [40]. Developing a diverse set of regulation strategies (e.g., peer engagement, physical exercise, breathing exercise) can improve an athlete’s ability to cope with emotional challenges [41]. Emotion regulation has also been linked to performance outcomes and may be strengthened through self-compassion [42].

Self-compassion refers to how individuals respond to themselves during moments of difficulty, such as perceived inadequacy or failure. It involves adopting an understanding and accepting stance toward oneself, characterized by mindfulness, nonjudgment, and recognition of shared human experience [43]. Practicing self-compassion entails extending the same care and support to oneself as one would to loved ones [44]. This capacity is shaped by early experiences and social relationships throughout development. Research has linked self-compassion to reduced psychopathology and enhanced motivation [45].

## 5. Sport Satisfaction

A student-athlete’s psychological well-being is influenced by their level of satisfaction with their sport [46], as their overall evaluation of their life often includes their experience as an athlete. Sport satisfaction is a positive affective state that results from a complex evaluation of structures, processes, and outcomes associated with the athletic experience [47]. This satisfaction can serve as a protective factor, helping to buffer against negative outcomes such as burnout [48].

An athlete’s overall satisfaction reflects the extent to which they feel content with their personal achievements, team accomplishments, and the support provided by their coaches [49]. Coaches can enhance sport satisfaction by addressing athletes’ immediate needs such as interpersonal conflict, performance challenges, and academic stress through tangible, informational, emotional, and esteem-based support [50]. Tangible support involves assistance with injuries and financial concerns; informational support includes guidance on techniques and strategies; emotional support refers to empathy and understanding; and esteem support focuses on reinforcing an athlete’s sense of self-worth [51].

Athletes may also experience motivation and a sense of belonging through relationships with coaches and teammates, as well as through engagement with the campus community [52,53]. The quality of the coach-athlete relationship depends on the athlete’s perception of its benefits (e.g., insight, support) and costs (e.g., pressure, loss of privacy) [54]. When athletes perceive shared values and experience a comfortable exchange of resources, they are more likely to develop stronger emotional connections with their team and the broader sport community [55]. Coaches who satisfy their own psychological needs, maintain high-quality coaching motivation, and achieve psychological well-being are more likely to develop supportive environments for effective athlete development [56]. Positive relationships with coaches and teammates foster relatedness and self-worth, which in turn enhance overall sport satisfaction [57].

## 6. The Present Study

The purpose of this study is to examine the presence of grit, growth mindset, emotion regulation, self-compassion, and psychological well-being among collegiate athletes, as well as the role these attributes play in their satisfaction with their sport experience. Specifically, this study investigates how athletes’ communities of upbringing (i.e., rural, suburban, urban) and their status within their sport (i.e., year in college and scholarship status) are associated with sport satisfaction, as well as differences in adaptive psychological attributes across sports. We propose the following research questions:

*Research Question 1:* How are athletes’ community of upbringing, year in college, and scholarship status associated with their sport satisfaction?

*Research Question 2:* What differences exist in adaptive psychological attributes across sports?

*Research Question 3:* To what extent does growth mindset moderate the relationship between psychological well-being and sport satisfaction?

## 7. Materials and Methods

### 7.1. Participants and Procedure

We recruited 263 participants of emerging adulthood age (18–29) [58] using CloudResearch, an online research recruitment service, as well as email outreach to directors of university athletic programs. The sample included 147 men (55.9%), 112 women (42.6%), and 4 transgender individuals (1.5%). Participants’ ages ranged from 18 years (3%) to 29 years (7.6%), with an average age of 24.19 years (*SD* = 2.97). Racial/ethnic identities among participants included American Indian (3.1%), Black (34.5%), seven variations of Biracial (13.4%), Asian (1.5%), Hispanic/Latine (9.6%), and White (37.9%). Communities of upbringing included rural (18.3%), suburban (45.2%), and urban (36.5%), with representation from 43 states and Washington, D.C. Participants attended public (84.4%) and private (15.6%) universities and colleges. A total of 89 participants (33.8%) reported having received a collegiate athletic scholarship, and 79 (30%) participants were currently receiving one. Of those who had ever received a scholarship, 53 reported receiving a full scholarship, while 36 reported receiving a partial scholarship.

Inclusion criteria required participants to be current collegiate student-athletes within the emerging adulthood age range and actively participating in an official university sport involving intercollegiate competition. Participants were provided with a consent form and anonymously completed a voluntary Qualtrics questionnaire approved by the institutional review board. The self-report questionnaire included items assessing personal demographics (i.e., age, gender, race/ethnicity, community of upbringing, year in college), grit, growth mindset, emotion regulation (i.e., cognitive reappraisal, expressive suppression), self-compassion, psychological well-being, and sport satisfaction. Descriptive statistics are available in Table 1 and correlations in Table 2.

### 7.2. Measures

***Satisfaction Scale for Athletes*** (SSA) [47]. The SSA is a 16-item instrument that measures how an individual feels about participating in their sports team. The instrument is measured on a 7-point scale ranging from 1 = *not at all satisfied* to 7 = *extremely satisfied*. The subscales include satisfaction with coach, satisfaction with team performance, and satisfaction with teammates. Sample items include the extent to which athletes are satisfied with “the manner in which my talents are (were) employed” and “the tactics used during games”. In the present study, the SSA achieved a Cronbach’s alpha coefficient of 0.94.

***World Health Organization-Five Well-Being Index*** (WHO-5) [59]. The WHO-5 is a 5-item instrument created by the World Health Organization with the goal of measuring an individual’s psychological well-being. The instrument is measured on a 6-point scale ranging from 0 = *at no time* to 5 = *all of the time*. Sample items include “Over the past two weeks, I have felt cheerful and in good spirits” and “Over the past two weeks, my daily life has been filled with things that interest me.” In the present study, the WHO-5 achieved a Cronbach’s alpha coefficient of 0.87.

***Short Grit Scale*** (Grit-S) [24]. The Grit-S Scale is an 8-item instrument measuring grit (perseverance and passion for long-term goals) on a 5-point scale ranging from 1 = *very much like me* to 5 = *not like me at all*. Four items are reverse scored, and higher total scores indicate higher scores of grit. The Grit-S scale includes items like “Setbacks don’t discourage me” and “I often set a goal but later choose to pursue a different one.” In the present study, the Grit-S scale achieved a Cronbach’s alpha coefficient of 0.54, with the perseverance of effort and consistency of interest subscales achieving Cronbach’s alpha coefficients of 0.63 and 0.74, respectively.

***Implicit Self-Theory Scale*** (ISTS) [60]. The ISTS is an 8-item instrument that contains two subscales (fixed and growth mindset), each with four items on a 6-point scale ranging from 1 = *strongly disagree* to 6 = *strongly agree*. This study utilizes the growth mindset scale, which includes items such as “I believe I can always substantially improve my intelligence” and “With enough time and effort I think I could significantly improve my intelligence level.” In this study, growth mindset achieved a Cronbach’s alpha coefficient of 0.86.

***Emotion Regulation Questionnaire*** (ERQ) [61]. The ERQ is a 10-item instrument that measures an individual’s ability to regulate their emotions on a 7-point scale ranging from 1 = *strongly disagree* to 7 = *strongly agree*. The ERQ includes two independent scales, cognitive reappraisal and expressive suppression, and does not yield a total score. Sample items include “When I’m faced with a stressful situation, I make myself think about it in a way that helps me stay calm” and “I keep my emotions to myself.” In the present study, cognitive reappraisal and expressive suppression achieved Cronbach’s alpha coefficients of 0.86 and 0.69, respectively.

***Self-Compassion Scale-Short Form*** (SCS-SF) [62]. The SCS-SF is a 12-item instrument that measures an individual’s ability to show themselves kindness and treat themselves nonjudgmentally. The instrument is measured on a 5-point scale ranging from 1 = *almost never* to 5 = *almost always.* Sample items include “I’m disapproving and judgmental about my own flaws and inadequacies” and “I try to be understanding and patient towards those aspects of my personality I don’t like.” In the present study, the SCS-SF achieved a Cronbach’s alpha coefficient of 0.67.

### 7.3. Data Analysis

Participants missing more than 10% of the scales were excluded (*n* = 21) [63]. Mean substitution imputation using the variable series mean was utilized to replace the remaining missing data, preserving the overall mean estimate of the variables [64,65]. The sport categories were dummy coded, with wrestling withheld as a reference in regression analyses. Growth mindset and psychological well-being were grand-mean centered in the regression analysis. Analyses were conducted using IBM SPSS Statistics V31. Preliminary analyses were conducted to ensure data did not violate assumptions of normality, linearity, homoscedasticity, and multicollinearity. A power analysis (G*Power 3.1) was conducted using an alpha of 0.05 and a power of 0.80, which indicated a sample size of approximately 260 was required to achieve a medium effect size [66]. To address the research questions, we conducted correlation analyses, chi-square tests, independent-samples *t* tests, analyses of variance (ANOVA), an analysis of covariance (ANCOVA), multiple linear regression, and a three-stage hierarchical linear regression.

## 8. Results

### 8.1. Community of Upbringing, Year in College, and Scholarship Status

An ANOVA revealed a significant difference in sport satisfaction across communities of upbringing, *F*(2, 258) = 5.06, *p* = 0.007, η^2^ = 0.04, with significant differences in satisfaction with coaches (*p* = 0.003), team performance (*p* = 0.038), and teammates (*p* = 0.022). Athletes from urban communities reported the highest levels of satisfaction with their coaches, team performance, and teammates, while those from suburban communities reported the lowest satisfaction in all three areas. Similarly, there was a significant difference in growth mindset across communities of upbringing, *F*(2, 260) = 4.16, *p* = 0.017, η^2^ = 0.03. Students from suburban communities reported significantly lower (*p* = 0.037) growth mindset compared to others, while students from urban communities reported significantly higher (*p* = 0.004) growth mindset.

When controlling for age, an ANCOVA revealed a significant difference in sport satisfaction across years in college, *F*(5, 252) = 3.18, *p* = 0.008, η^2^ = 0.06. Fourth-year college athletes reported the highest sport satisfaction (*M* = 93.28, *SD* = 14.09), whereas seventh-year athletes reported the lowest (*M* = 66.00, *SD* = 15.56), followed by first-year athletes (*M* = 82.57, *SD* = 17.30). A chi-square test of independence was conducted to examine the relationship between receiving an athletic scholarship and year in college, and this relationship was significant, χ^2^(2, *N* = 236) = 15.36, *p* = 0.009, *V* = 0.24, with athletes in their second year reporting the most scholarships, followed by athletes in their third year.

An independent-samples *t* test showed a significant difference in sport satisfaction between athletes who received a full versus partial scholarship, *t*(87) = 3.94, *p* < 0.001, *d* = 0.85. There was no significant difference in sport satisfaction between athletes who received any scholarship and those who did not. However, a significant difference in sport satisfaction was found between athletes who had ever received a full athletic scholarship and those who had never received any scholarship, *t*(222) = 2.91, *p* = 0.004, *d* = 0.46, with student-athletes who received a full scholarship reporting significantly higher sport satisfaction. We also found a significant difference in sport satisfaction between those who received a partial scholarship and those who had never received an athletic scholarship, *t*(205) = 2.20, *p* = 0.029, *d* = 0.40, with those who had never received an athletic scholarship reporting significantly higher sport satisfaction than those who received a partial scholarship.

A significant difference in expressive suppression was found between those who had received an athletic scholarship and those who had not, *t*(261) = 2.36, *p* = 0.019, *d* = 0.31, with those who had received a scholarship reporting significantly greater expressive suppression. There was no significant difference in cognitive reappraisal between those who had and had not received an athletic scholarship (*p* = 0.064). We found a significant difference in grit, *t*(87) = 2.14, *p* = 0.035, *d* = 0.46, and self-compassion, *t*(87) = 2.32, *p* = 0.023, *d* = 0.50, between those who had received a full versus partial athletic scholarship, with those who received a full scholarship reporting significantly greater grit and self-compassion. Student-athletes who received a full scholarship reported significantly greater perseverance of effort (*p* = 0.022, *d* = 0.35) than all other participants, with no significant differences in consistency of interest.

### 8.2. Differences Across Sports

Differences in adaptive psychological attributes across sports were examined using a series of multiple regressions. Analysis revealed a significant difference in growth mindset across sports, *F*(29, 233) = 1.52, *p* = 0.049, *R*^2^ = 0.16. Fencing (B = −5.87, 95% CI [−10.38, −1.37], *β* = −0.17, *p* = 0.011), tennis (B = −2.68, 95% CI [−4.96, −0.41], *β* = −0.16, *p* = 0.021), ultimate frisbee (B = −7.22, 95% CI [−11.93, −2.51], *β* = −0.24, *p* = 0.003), and water polo (B = −8.60, 95% CI [−15.81, −1.40], *β* = −0.14, *p* = 0.020) were significant predictors of growth mindset. Across sports, analyses indicated no significant differences in grit (*p* = 0.15), self-compassion (*p* = 0.16), cognitive reappraisal (*p* = 0.94), expressive suppression (*p* = 0.72), well-being (*p* = 0.18), or sport satisfaction (*p* = 0.40).

### 8.3. Predicting Sport Satisfaction

**Figure 1:**
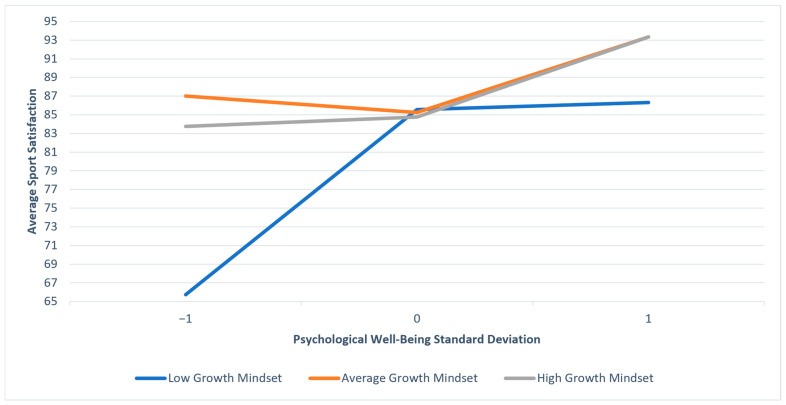
Interaction Effect of Growth Mindset and Psychological Well-Being on Sport Satisfaction.

We ran a three-stage hierarchical linear regression with sport satisfaction as the dependent variable. We entered grit, growth mindset, emotion regulation (i.e., cognitive reappraisal, expressive suppression), and self-compassion at stage one; well-being at stage two; and the interaction between well-being and growth mindset at stage three. Regression statistics are available in Table 3, and Figure 1 illustrates the interaction effect of growth mindset and psychological well-being on sport satisfaction.

The hierarchical regression revealed at stage 1 that growth mindset, cognitive reappraisal, expressive suppression, and self-compassion significantly contributed to the model: *F*(5, 255) = 26.37, *p* < 0.001, *R*^2^ = 0.34. Grit did not significantly contribute to the model at stage 1. Introducing well-being to the model at stage 2 explained an additional 9% of the variance in sport satisfaction, and this change was significant: *F*(6, 254) = 32.53, *p* < 0.001, *R*^2^ = 0.43. Self-compassion, cognitive reappraisal, and expressive suppression no longer significantly contributed to the model at stage 2. Introducing the interaction between growth mindset and well-being at stage 3 explained an additional 1% of the variance in sport satisfaction, and this change was significant: *F*(7, 253) = 28.84, *p* = 0.033, *R*^2^ = 0.44.

## 9. Discussion

This study explored how athletes’ identities and experiences relate to their adaptive psychological attributes and sport satisfaction. The findings extend the sport satisfaction literature by demonstrating how factors such as athlete identity, upbringing, scholarship status, and psychological attributes are associated with overall sport experience. Differences in both sport satisfaction and growth mindset across communities of upbringing suggest that early environmental context may play a meaningful role in shaping athletes’ psychological development and sport experiences [14,67,68]. Athletes from urban backgrounds tended to report more favorable perceptions of their sport environment, whereas those from suburban backgrounds reported comparatively lower satisfaction and growth-oriented beliefs. These patterns may reflect differences in access to competitive opportunities, exposure to adversity, and the availability of social and developmental resources across communities [13,69].

Athletes’ year in college was significantly associated with sport satisfaction, with satisfaction increasing from the first through the fourth year and declining thereafter. Year in college was also related to the likelihood of receiving an athletic scholarship, with fully funded athletes reporting the highest satisfaction. Student-athletes in later academic years have historically reported higher quality of college life and greater playing time, and increased playing time has been associated with higher levels of gratitude and satisfaction with the athletic experience [70,71].

Financial compensation is often viewed as an extrinsic motivator and is sometimes assumed to reduce intrinsic satisfaction. However, prior research [72,73] has produced mixed findings regarding the relationship between athletic scholarships and sport satisfaction. The present study supports evidence that well-compensated athletes may experience greater satisfaction. Importantly, the findings suggest that the type of scholarship matters. Athletes receiving partial scholarships reported significantly lower sport satisfaction than both fully funded athletes and those without scholarships.

This pattern may reflect differences in perceived security and pressure. Research indicates that many student-athletes believe they would be unable to afford tuition without scholarship support [2]. Scholarship status has also been linked to anxiety levels, with lower funding associated with greater anxiety and effort-related pressure [74,75]. Furthermore, athletes receiving full scholarships have been found to report higher satisfaction, perceived competence, and a stronger sense that their skills are utilized compared to those receiving partial scholarships [76]. In this study, participants who received a full scholarship reported significantly greater self-compassion than partially funded student-athletes and significantly greater perseverance in their efforts than those who received partial or no scholarships. These findings may reflect the additional effort and self-compassionate attitude student-athletes may need to cultivate to obtain a full athletic scholarship.

Mixed results were observed across sports, with the most notable differences emerging in growth mindset. Athletes in fencing, tennis, ultimate frisbee, and water polo reported a significantly lower growth mindset compared to athletes in other sports. Teams aiming to foster a growth mindset may benefit from cultivating environments that emphasize positivity and mastery orientation. Research [77] indicates that positive sport climates support the development of a growth mindset, which is associated with improved athletic performance, particularly among elite athletes [19].

The findings also indicate that grit, growth mindset, emotion regulation, and self-compassion are significantly associated with psychological well-being among student-athletes, with cognitive reappraisal showing the strongest relationship and expressive suppression the weakest. Previous research [78] has demonstrated that effective emotion regulation is linked to positive psychological functioning, whereas maladaptive emotion regulation strategies can impair cognitive processes. Growth mindset showed the strongest association with sport satisfaction, whereas expressive suppression showed the weakest. Scholars have suggested that athletes who utilize cognitive reappraisal are likely to experience greater satisfaction in their sport experience, while those who rely on expressive suppression tend to experience fewer positive emotions and greater mental health challenges [79]. In this study, expressive suppression was positively associated with sport satisfaction, indicating that a level of restraint among student-athletes may support their sport experience. Furthermore, results extend previous research that has found growth mindset to be associated with job satisfaction and life satisfaction [80,81].

The interaction between well-being and growth mindset in predicting sport satisfaction suggests that belief in one’s capacity to grow may influence the impact of psychological well-being on athletic experience. Although the interaction effect was statistically significant, its contribution to the model was small. As illustrated in Figure 1, this limited explanatory power may reflect particularly low satisfaction among athletes with both a low growth mindset and low psychological well-being, whereas those with an average to high growth mindset reported similar levels of satisfaction across average and high levels of psychological well-being. Student-athletes with a low growth mindset and low psychological well-being appear especially vulnerable to poorer sport experiences. While growth mindset contributes meaningfully to sport satisfaction in this study, psychological well-being may serve as a more substantial driver. Accordingly, interventions that target both mental health and growth-oriented thinking may effectively enhance athletes’ satisfaction. Supporting psychological well-being may provide a foundation upon which growth mindset can further enrich the sport experience.

More broadly, athletes appear to benefit not only from maintaining psychological well-being but also from cultivating a belief in their ability to improve. Research has shown that teams emphasizing personal improvement report higher athlete satisfaction than those focused primarily on outperforming others [82]. Athletes with a stronger growth mindset are also more likely to adopt adaptive coping strategies when facing challenges, contributing to greater satisfaction [35]. Additionally, growth mindset has been linked to greater effort investment [83], and higher effort has been associated with increased sport satisfaction [84].

## 10. Limitations

This study has several limitations. First, representation across sports was uneven. While some sports (e.g., basketball, football, and soccer) were well represented, others (e.g., water polo, ice hockey, and rugby) had relatively few participants, which may limit sport-specific conclusions. Second, the study population was primarily composed of Black and White participants, with limited representation of other ethnic groups, which limits the generalizability of the results across identities. Third, the Grit-S scale demonstrated a low Cronbach’s alpha coefficient, limiting the reliability of grit-related findings. Fourth, the cross-sectional design limits causal inference and does not capture changes in athletes’ experiences over time. Despite these limitations, the study provides meaningful contributions to understanding the relationships among athletes’ mindset, mental health, and sport experience.

## 11. Conclusions

Student-athletes face a range of pressures that can intensify the mental health challenges commonly associated with academic life. The present findings offer several important implications for stakeholders invested in student-athlete well-being and performance. In particular, these results can inform the development of targeted interventions aimed at enhancing both sport satisfaction and psychological well-being, as well as future longitudinal studies that seek to investigate how athletes’ experiences over time influence adaptive psychological attributes. It is recommended that coaching staff consider how factors such as community of upbringing, year in college, and scholarship status may influence athletes’ satisfaction and well-being. By fostering greater awareness of these dynamics, parents and coaches can adopt more intentional and effective practices to better support athletes. Additionally, emphasizing the development of adaptive psychological attributes such as grit, growth mindset, emotion regulation, and self-compassion may play a critical role in helping student-athletes navigate the unique challenges of academics and athletics.

## Figures and Tables

**Table 1:** Descriptive Statistics (*N* = 263).

	Variables	*n*	*M* Grit (*SD*)	*M* Growth Mindset (*SD*)	*M* Cognitive Reappraisal (*SD*)	*M* Expressive Suppression (*SD*)	*M* Self-Compassion (*SD*)	*M* Well-Being (*SD*)	*M* Sport Satisfaction (*SD*)
Gender	Men	147	27.85 (*4.69*)	20.00 (*3.45*)	31.60 (*6.43*)	19.16 (*4.69*)	38.09 (*5.42*)	22.16 (*5.39*)	88.80 (*15.22*)
	Women	112	26.96 (*4.72*)	21.13 (*5.31*)	30.76 (*7.10*)	17.51 (*5.09*)	36.65 (*7.35*)	21.13 (*5.31*)	86.59 (*16.37*)
Community of Upbringing	Rural	48	26.94 (*5.08*)	19.19 (*3.93*)	30.17 (*7.31*)	17.85 (*5.17*)	37.44 (*6.41*)	21.31 (*5.48*)	87.00 (*15.84*)
	Suburban	119	26.85 (*4.09*)	19.08 (*3.70*)	31.14 (*6.36*)	18.51 (*4.49*)	36.76 (*6.12*)	20.97 (*5.11*)	85.10 (*15.37*)
	Urban	96	28.29 (*5.20*)	20.47 (*3.55*)	31.98 (*6.86*)	18.46 (*5.45*)	38.12 (*6.66*)	22.92 (*5.41*)	91.79 (*15.21*)
Scholarship Status	Full Scholarship	53	28.32 (*5.06*)	19.89 (*3.79*)	33.56 (*7.01*)	19.78 (*5.20*)	38.73 (*6.84*)	22.70 (*5.71*)	94.11 (*14.72*)
	Partial Scholarship	36	26.17 (*3.99*)	19.28 (*3.25*)	30.78 (*7.37*)	19.00 (*5.10*)	35.58 (*5.38*)	20.44 (*5.10*)	81.08 (*16.17*)
	No Scholarship	174	27.38 (*4.74*)	19.56 (*3.82*)	30.72 (*6.41*)	17.86 (*4.84*)	37.30 (*6.36*)	21.67 (*5.23*)	87.25 (*15.10*)
Year in School	First Year	58	27.52 (*4.73*)	19.16 (*3.98*)	30.14 (*6.71*)	17.28 (*5.15*)	36.66 (*5.51*)	21.53 (*5.70*)	82.57 (*17.30*)
	Second Year	88	27.18 (*4.88*)	18.94 (*3.87*)	30.58 (*6.61*)	18.10 (*4.85*)	37.88 (*6.28*)	21.27 (*5.04*)	88.38 (*13.76*)
	Third Year	56	27.54 (*5.15*)	20.00 (*3.59*)	31.96 (*6.18*)	18.86 (*4.44*)	36.69 (*7.39*)	21.52 (*5.26*)	89.52 (*14.49*)
	Fourth Year	40	27.93 (*4.14*)	20.85 (*3.03*)	33.10 (*6.90*)	19.88 (*4.93*)	38.65 (*6.42*)	23.78 (*5.24*)	93.28 (*14.09*)
	Fifth Year	19	26.32 (*4.10*)	20.11 (*3.59*)	32.11 (*7.56*)	18.68 (*6.04*)	36.37 (*5.87*)	20.89 (*5.69*)	88.00 (*19.94*)
	Seventh Year	2	28.50 (*7.78*)	21.00 (*4.24*)	30.50 (*14.85*)	16.00 (*5.66*)	39.50 (*9.19*)	22.00 (*7.07*)	66.00 (*15.56*)
Sport	Air Rifle	7	25.86 (*2.91*)	20.57 (*3.78*)	35.29 (*5.09*)	21.43 (*4.43*)	37.76 (*4.42*)	19.71 (*6.18*)	85.14 (*14.67*)
	Baseball	34	26.56 (*3.98*)	19.15 (*3.89*)	31.47 (*7.25*)	19.15 (*5.80*)	37.74 (*5.31*)	22.53 (*5.02*)	92.44 (*15.40*)
	Basketball	115	25.57 (*4.78*)	20.01 (*3.55*)	31.46 (*6.80*)	18.73 (*4.96*)	38.15 (*6.22*)	21.79 (*5.56*)	87.77 (*15.65*)
	Beach Volleyball	8	25.25 (*4.20*)	20.25 (*3.88*)	30.00 (*8.38*)	19.63 (*6.12*)	39.92 (*6.90*)	23.88 (*4.39*)	89.62 (*20.13*)
	Bowling	15	24.47 (*2.00*)	18.33 (*4.01*)	30.53 (*6.65*)	18.60 (*6.00*)	36.13 (*3.11*)	20.73 (*4.30*)	87.27 (*16.39*)
	Cheerleading	20	27.40 (*4.85*)	19.50 (*3.97*)	32.30 (*7.22*)	19.90 (*3.95*)	39.30 (*6.74*)	24.65 (*3.80*)	89.65 (*16.69*)
	Cross Country Running	9	29.67 (*7.68*)	20.00 (*3.74*)	30.56 (*10.75*)	19.00 (*4.00*)	39.44 (*10.05*)	19.00 (*6.71*)	90.11 (*10.48*)
	Dance	26	26.88 (*5.09*)	19.08 (*4.33*)	31.92 (*6.23*)	19.27 (*5.98*)	38.27 (*6.09*)	23.12 (*4.53*)	94.62 (*13.50*)
	E-Sports	16	26.31 (*5.02*)	19.69 (*3.50*)	30.69 (*5.75*)	18.50 (*5.74*)	37.38 (*6.67*)	21.13 (*5.08*)	90.75 (*9.13*)
	Fencing	3	24.67 (*0.58*)	15.00 (*6.08*)	28.67 (*5.69*)	15.33 (*5.69*)	34.67 (*2.31*)	21.00 (*4.36*)	83.33 (*12.50*)
	Field Hockey	2	24.00 (*0.00*)	21.00 (*4.24*)	32.00 (*9.90*)	22.00 (*8.49*)	44.50 (*13.44*)	23.50 (*9.19*)	86.00 (*36.77*)
	Football	48	27.46 (*5.07*)	19.54 (*3.19*)	31.88 (*6.08*)	19.44 (*4.52*)	38.02 (*6.70*)	23.08 (*4.71*)	90.98 (*16.46*)
	Golf	14	28.71 (*4.82*)	20.50 (*3.48*)	32.21 (*6.54*)	17.14 (*5.64*)	38.79 (*6.09*)	22.14 (*4.90*)	84.86 (*15.84*)
	Gymnastics	10	26.90 (*4.68*)	19.40 (*3.37*)	30.80 (*4.26*)	19.20 (*3.74*)	35.40 (*5.34*)	20.70 (*4.24*)	84.80 (*22.38*)
	Ice Hockey	3	25.33 (*0.58*)	16.67 (*1.15*)	26.33 (*2.31*)	18.00 (*3.46*)	35.67 (*1.15*)	23.67 (*3.05*)	88.67 (*10.79*)
	Ice Skating	2	24.00 (*5.67*)	19.00 (*1.41*)	31.00 (*4.24*)	21.50 (*7.78*)	34.50 (*2.12*)	21.50 (*0.71*)	76.00 (*8.49*)
	Lacrosse	4	28.50 (*4.36*)	20.50 (*4.36*)	29.50 (*9.29*)	19.00 (*1.83*)	36.50 (*8.89*)	20.50 (*5.32*)	87.75 (*8.14*)
	Martial Arts	6	30.17 (*7.08*)	22.67 (*2.16*)	33.83 (*7.70*)	21.00 (*6.65*)	42.00 (*9.47*)	23.50 (*6.35*)	97.50 (*11.79*)
	Nordic Skiing	2	24.00 (*1.41*)	23.00 (*1.41*)	32.50 (*7.78*)	25.00 (*0.00*)	34.50 (*4.95*)	25.50 (*2.12*)	84.50 (*2.12*)
	Rowing	2	25.00 (*0.00*)	21.00 (*4.24*)	39.00 (*1.41*)	21.50 (*4.95*)	35.00 (*4.24*)	19.00 (*7.07*)	78.50 (*10.61*)
	Rugby	2	28.00 (*4.24*)	19.00 (*5.66*)	31.00 (*2.83*)	16.50 (*3.54*)	38.50 (*3.54*)	24.00 (*4.24*)	78.50 (*3.53*)
	Soccer	36	27.59 (*4.55*)	19.58 (*3.22*)	31.83 (*6.57*)	18.97 (*4.70*)	38.39 (*6.15*)	23.41 (*4.01*)	87.67 (*15.29*)
	Softball	12	24.50 (*3.63*)	18.50 (*5.21*)	29.83 (*5.49*)	16.92 (*5.40*)	33.08 (*6.99*)	20.33 (*4.05*)	77.58 (*12.29*)
	Swim and Dive	8	26.25 (*4.27*)	18.75 (*3.69*)	29.50 (*5.29*)	21.13 (*3.87*)	35.88 (*3.94*)	21.13 (*5.64*)	84.29 (*16.96*)
	Tennis	13	25.77 (*3.75*)	17.46 (*3.20*)	29.00 (*7.52*)	19.69 (*5.50*)	35.62 (*3.52*)	22.62 (*4.37*)	84.15 (*15.60*)
	Track and Field	34	29.06 (*4.30*)	19.85 (*3.73*)	31.91 (*7.18*)	18.50 (*4.17*)	37.65 (*6.41*)	21.73 (*5.64*)	89.06 (*12.85*)
	Ultimate Frisbee	4	23.50 (*2.38*)	14.75 (*3.40*)	26.75 (*8.85*)	18.25 (*0.50*)	35.50 (*5.26*)	16.50 (*3.32*)	73.25 (*15.20*)
	Volleyball	30	25.93 (*3.85*)	19.27 (*3.90*)	30.53 (*5.52*)	18.13 (*4.96*)	34.07 (*5.37*)	20.10 (*4.74*)	86.67 (*14.59*)
	Water Polo	1	26.00 (*-*)	11.00 (*-*)	28.00 (*-*)	17.00 (*-*)	33.00 (*-*)	14.00 (*-*)	63.00 (*-*)
	Wrestling	10	27.20 (*4.80*)	18.80 (*3.43*)	31.10 (*6.73*)	21.50 (*4.06*)	39.50 (*7.29*)	23.30 (*4.76*)	94.60 (*14.07*)

**Table 2:** Correlations between Grit, Growth Mindset, Cognitive Reappraisal, Expressive Suppression, Self-Compassion, Well-Being, and Sport Satisfaction (*N* = 263).

Variables	Grit	Growth Mindset	Cognitive Reappraisal	Expressive Suppression	Self-Compassion	Well-Being	Sport Satisfaction
Grit	-						
Growth Mindset	0.369 ***	-					
Cognitive Reappraisal	0.341 ***	0.501 ***	-				
Expressive Suppression	−0.015	0.045	0.260 ***	-			
Self-Compassion	0.473 ***	0.310 ***	0.464 ***	0.027	-		
Well-Being	0.378 ***	0.322 ***	0.427 ***	0.174 **	0.383 ***	-	
Sport Satisfaction	0.273 ***	0.507 ***	0.466 ***	0.190 **	0.338 ***	0.533 ***	-

** = *p* < 0.01. *** = *p* < 0.001.

**Table 3:** Hierarchical Regression Analysis Showing Grit, Growth Mindset, Cognitive Reappraisal, Expressive Suppression, Self-Compassion, and Well-Being as Predictors of Sport Satisfaction.

Variables	*B*	*SE B*	*β*	*t*	95% CI	*r*	*r* ^2^	*sr* ^2^	*F*-Change	*R* ^2^	*R*^2^ Change
Step 1									26.37 ***	0.34	0.34
(Constant)	54.04	6.85		7.89 ***							
Grit	0.07	0.20	0.02	0.33	[−0.33, 0.46]	0.27	0.02	0.02			
Growth Mindset	1.50	0.25	0.36	5.92 ***	[1.00, 2.01]	0.51	0.35	0.30			
Cognitive Reappraisal	0.44	0.15	0.19	2.86 **	[0.14, 0.74]	0.47	0.18	0.15			
Expressive Suppression	0.38	0.17	0.12	2.26 *	[0.05, 0.71]	0.19	0.14	0.12			
Self-Compassion	0.30	0.15	0.12	1.98 *	[0.002, 0.61]	0.34	0.12	0.10			
Step 2									42.07 ***	0.43	0.09
(Constant)	74.11	7.07		10.48 ***							
Grit	−0.16	0.19	−0.05	−0.87	[−0.53, 0.21]	0.27	−0.06	−0.04			
Growth Mindset	1.39	0.24	0.33	5.86 ***	[0.92, 1.85]	0.51	0.35	0.28			
Cognitive Reappraisal	0.26	0.15	0.11	1.81	[−0.02, 0.55]	0.47	0.11	0.09			
Expressive Suppression	0.25	0.16	0.08	1.57	[−0.06, 0.55]	0.19	0.10	0.07			
Self-Compassion	0.15	0.14	0.06	1.02	[−0.14, 0.43]	0.34	0.06	0.05			
Well-Being	1.06	0.16	0.36	6.49 ***	[0.73, 1.38]	0.53	0.38	0.31			
Step 3									4.24 *	0.44	0.01
(Constant)	71.33	7.16		9.97 ***							
Grit	−0.15	0.19	−0.05	−0.82	[−0.52, 0.21]	0.27	−0.05	−0.04			
Growth Mindset	3.04	0.84	0.73	5.33 ***	[0.81, 1.76]	0.51	0.32	0.25			
Cognitive Reappraisal	0.28	0.15	0.12	1.96	[−0.001, 0.57]	0.47	0.12	0.09			
Expressive Suppression	0.26	0.16	0.08	1.64	[−0.05, 0.56]	0.19	0.10	0.08			
Self-Compassion	0.21	0.15	0.09	1.41	[−0.08, 0.49]	0.34	0.09	0.07			
Well-Being	2.65	0.79	0.91	6.58 ***	[0.75, 1.38]	0.53	0.38	0.31			
Growth Mindset and Well-Being Interaction	−0.08	0.04	−0.79	−2.06 *	[−0.16, −0.003]	−0.07	−0.13	−0.10			

* = *p* < 0.05. ** = *p* < 0.01. *** = *p* < 0.001.

## Data Availability

The data associated with this study is publicly available and can be accessed at https://doi.org/10.7910/DVN/IQSSOU (accessed on 12 April 2026).

## Abbreviations

The following abbreviations are used in this manuscript:

NCAA	National Collegiate Athletics Association
SSA	Satisfaction Scale for Athletes
WHO-5	World Health Organization-Five Well-Being Index
Grit-S	Short Grit Scale
ISTS	Implicit Self-Theory Scale
ERQ	Emotion Regulation Questionnaire
